# A Rare Incidence of Hepatocellular Carcinoma With Tumor Thrombus Extending to the Right Heart

**DOI:** 10.7759/cureus.43965

**Published:** 2023-08-23

**Authors:** Said Amin, Aymen Shahab, Abdur Rahman Qazi, Hamza Yunus, Laraib Saeed, Asad Ali Khan, Mohammad Ebad Ur Rehman, Nisar Ahmad Khan

**Affiliations:** 1 Medicine, Ayub Teaching Hospital, Abbottabad, PAK; 2 Diagnostic Radiology, Hayatabad Medical Complex Peshawar, Peshawar, PAK; 3 Gastroenterology, Hayatabad Medical Complex Peshawar, Peshawar, PAK; 4 Internal Medicine, Hayatabad Medical Complex Peshawar, Peshawar, PAK; 5 Gastroenterology and Hepatology, Hayatabad Medical Complex Peshawar, Peshawar, PAK; 6 Medicine, Holy Family Hospital, Rawalpindi, PAK; 7 Internal Medicine, Lady Reading Hospital, Peshawar, PAK

**Keywords:** alpha-fetoprotein, inferior vena cava thrombosis, intracardiac thrombus metastasis, tumor thrombus metastasis, hepatocellular carcinoma

## Abstract

Hepatocellular carcinoma (HCC) is a leading cause of cancer-related deaths and the sixth most commonly diagnosed cancer worldwide due to several common risk factors, including hepatitis C virus (HCV), hepatitis B virus (HBV), and other causes of cirrhosis. In HCC, intrahepatic vascular invasion and a tumor thrombus are commonly observed. However, the extrahepatic spread of the tumor thrombus to the heart via the portal vein, hepatic vein, and inferior vena cava (IVC) is rarely reported and is considered a poor prognostic factor. In addition, rarely, there is a risk of cor pulmonale and thromboembolism of the pulmonary vessels. Our patient also presented with this rare complication of HCC.

Our patient’s clinical presentation was bilateral pedal edema, moderate ascites, and abdominal discomfort with raised jugular venous pressure. These signs and symptoms are related to an impairment of the right heart caused by intracardiac tumor thrombus metastasis, leading to diastolic dysfunction. Based on these findings, echocardiography and abdominal computed tomography (CT) scan were performed with the definitive diagnosis of hepatocellular carcinoma with tumor thrombus metastases in the hepatic vein, inferior vena cava, and right atrium. The management team agreed on a conservative treatment plan based on the advanced stage of the disease and the high risk associated with aggressive treatment modalities.

Unfortunately, on day 7 of admission, the patient died from a possible pulmonary embolism that led to cardiopulmonary arrest. This case underscores the importance of screening patients with a high HCC tumor burden with abdominal ultrasound and echocardiography for early detection and timely management.

## Introduction

Primary liver cancer is the fifth most frequently diagnosed cancer and the third leading cause of cancer-related mortality [1] worldwide after lung and colorectal cancer. In 2020, approximately 906,000 new cases and 830,000 deaths were reported due to liver cancer [2]. Globally, hepatocellular carcinoma (HCC) comprises around 75% of all primary liver cancer cases [3]. HCC affects males more frequently than females, with a 3:1 male-to-female ratio [4]. The five-year survival rate of HCC is approximately 18% [5].

The major risk factors for the development of HCC are liver cirrhosis from any cause. In one study, it was reported that 56% of the cases of HCC were attributable to the hepatitis B virus (HBV), while 20% were due to the hepatitis C virus (HCV) [6]. Other possible risk factors for HCC include nonalcoholic fatty liver disease (NAFLD), obesity, metabolic syndrome, diabetes mellitus, smoking, heavy alcohol intake, dietary aflatoxin B1 exposure, and inherited diseases such as hereditary hemochromatosis and alpha-1 antitrypsin deficiency.

HCC remains asymptomatic and undetected in most cases until the advanced stage. In some cases, there is vague right upper quadrant abdominal pain, a palpable abdominal mass, weight loss, jaundice, and rarely ascites. HCC metastasizes mainly to the lungs, abdominal lymph nodes, bone, and adrenal gland. Intracardiac tumor thrombus metastasis is quite rare, despite the increased tendency of HCC for vascular invasion and the location of the heart upstream from the liver through the inferior vena cava (IVC). In addition, Budd-Chiari syndrome has been even rarely associated with HCC and associated tumor thrombus formation [7]. Here, we present an interesting case of an elderly patient who initially presented with signs and symptoms of right heart failure with a component of hepatic pathology. Further investigations revealed markedly raised alpha-fetoprotein (AFP) levels and the presence of an enhancing intrahepatic lesion with tumor thrombus extension into the right heart on triphasic computed tomography (CT). The patient was diagnosed with HCC with tumor thrombus metastasis to the right heart and was put on a conservative palliative care regimen. This case emphasizes the unusual presentation of HCC and the importance of HCC surveillance in high-risk patients with abdominal ultrasound and transthoracic echocardiography in those with HCC so that the disease can be identified early.

## Case presentation

This 85-year-old male hypertensive patient presented to us in the emergency department with progressive shortness of breath for one week, abdominal distention and bilateral swelling of feet up to the ankles for 10 days, anorexia, and generalized body weakness for one month. Examination revealed an ill-looking male with a Glasgow Coma Scale (GCS) score of 15/15. His vital signs were as follows: pulse rate of 80 per minute (weak and regular), blood pressure (BP) of 175/105, respiratory rate of 18 breaths per minute, and oxygen saturation of 90% (room air). Of note, his jugular venous pulse (JVP) was measured up to 9 cm, and bilateral pedal edema was present; however, no peripheral stigmata of chronic liver disease were found.

A systemic examination of the patient revealed a moderately distended, non-tender abdomen with positive shifting dullness. His cardiovascular examination revealed normal S1 and S2 heart sounds and a grade II systolic murmur at the tricuspid area with a faint S3. Respiratory examination showed bilateral dullness on percussion and diminished breath sounds in the lower zones of the lungs. On examination, there were mixed signs of right heart failure and liver failure, including a raised JVP, ascites, dyspnea, systolic murmur, and bilateral pedal edema.

**Figure 1:**
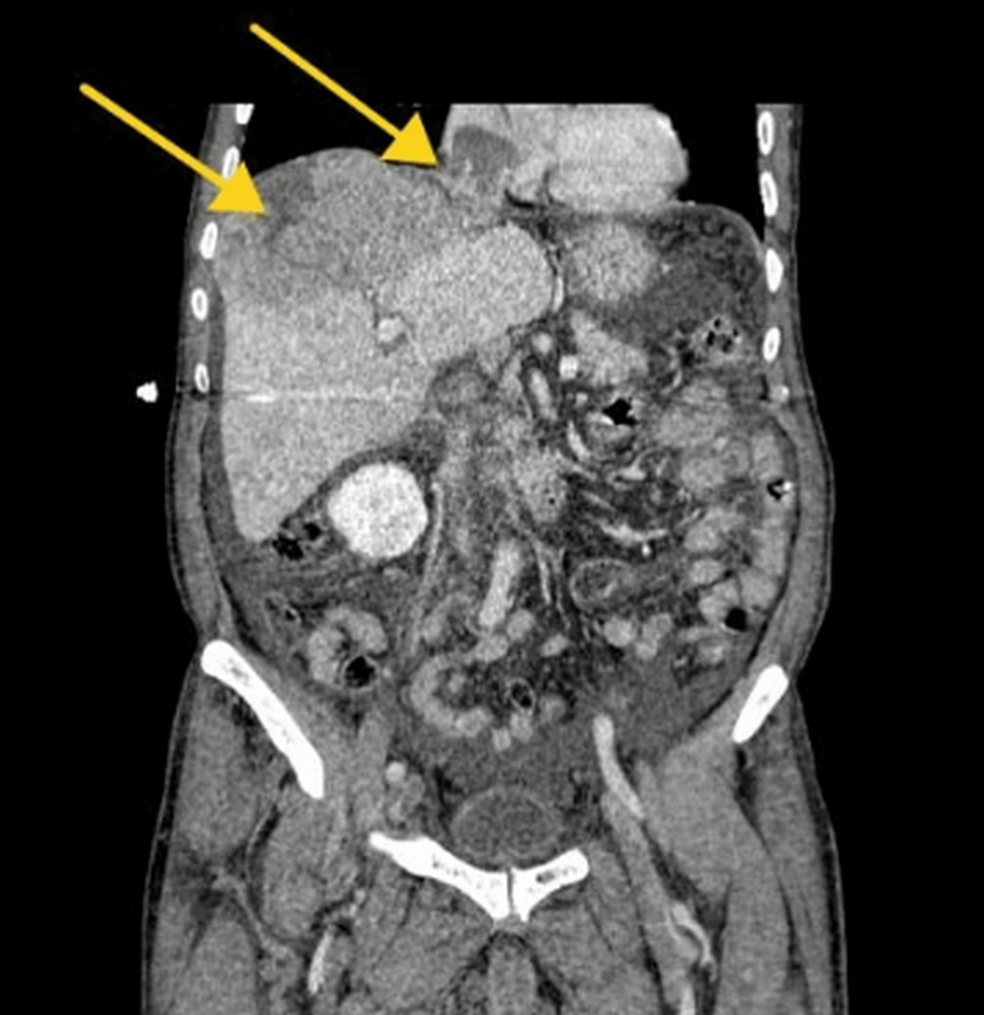
Dynamic hepatic CT scan porto-venous phase coronal reformat image revealing well-defined hypodense hepatic lesion in segments VII and VIII extending to involve the IVC and extending into the right atrium (arrows) CT: computed tomography, IVC: inferior vena cava

**Figure 2:**
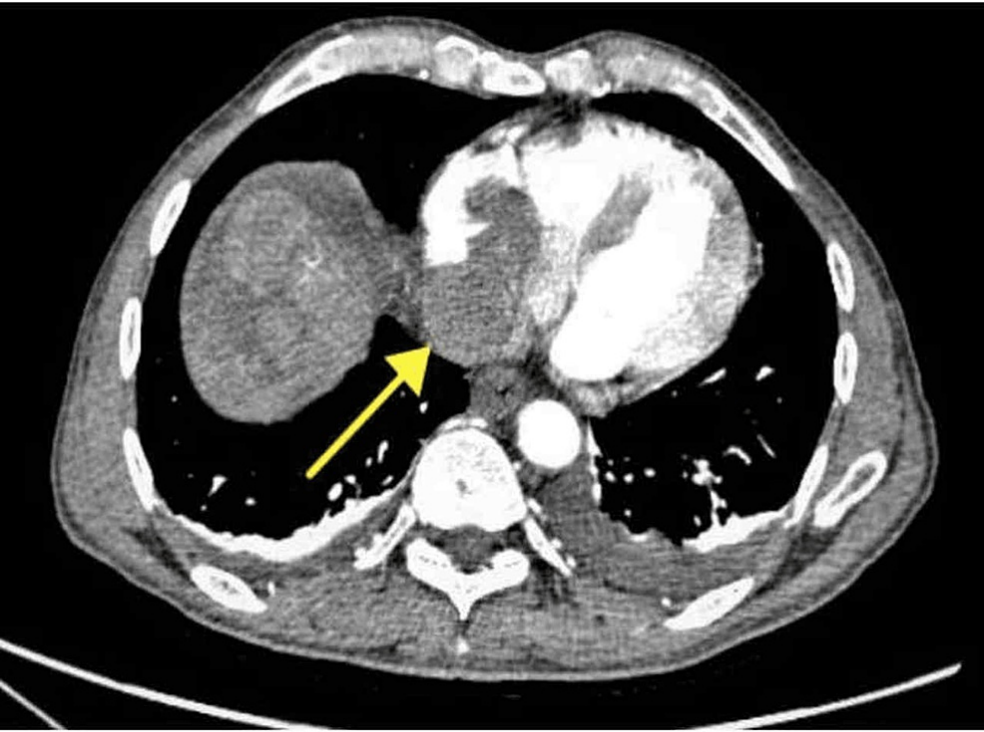
Dynamic hepatic CT scan axial section in arterial phase: included section of the chest at the level of the heart reveals a well-defined hypodense filling defect extending from the IVC in the right atrium (arrow) CT: computed tomography, IVC: inferior vena cava

**Figure 3:**
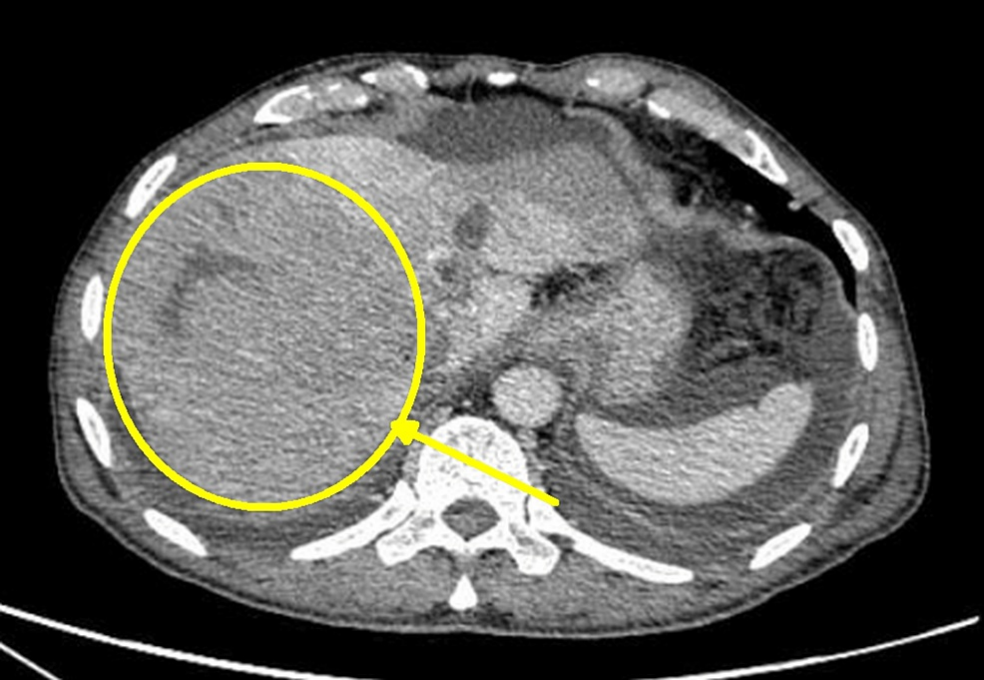
Dynamic hepatic CT scan revealing a large hypodense lesion in the right lobe of the liver causing compression of the right hepatic vein (arrow) CT: computed tomography

The patient’s abdominal ultrasound scan showed enlarged liver with a single lesion of approximately 128 × 87 mm in the right lobe of the liver along with moderate abdominal and pelvic ascites, while a Doppler study revealed internal vascularity of the mass lesion abutting the adjacent intrahepatic IVC, and an echogenic thrombus was observed at the confluence of hepatic veins, likely of tumor extension. The hepatic and suprahepatic IVC was dilated and showed a tumor thrombus extending to the right atrium with about a 4.2 × 3.7 intracardiac lesion (Figures 1, 2). A triphasic computed tomography (CT) scan of the abdomen revealed an enlarged liver measuring 19 cm and a hypodense lesion with internal necrosis occupying almost the whole of the right lobe, reaching the subcapsular region, and surrounding neovascularization (Figure 3). Thus, the CT scan report confirmed the presence of HCC with inferior vena cava (IVC) and intracardiac extension of tumor thrombus, along with moderate abdominopelvic ascites. On further workup, his alpha-fetoprotein (AFP) levels were found to be very high (>2,000 IU/L), and liver function tests (LFT) were also deranged (Table 1). Apart from raised D-dimers, slightly low fibrinogen levels, and raised fibrinogen degradation products, the rest of the coagulation profile, including protein C and protein S levels, factor V, antithrombin III, and homocysteine levels were normal (Table 1).

**Table 1:** Laboratory investigations AST: aspartate transaminase, ALT: alanine transaminase, HBV: hepatitis B virus, HCV: hepatitis C virus, HIV: human immunodeficiency virus, ELISA: enzyme-linked immunosorbent assay, PT: prothrombin time, INR: international normalized ratio, aPTT: activated partial thromboplastin time, FDP: fibrinogen degradation products

Laboratory investigation	Name of test	Values
Tumor marker	Alpha-fetoprotein	>2,000 IU/L
Liver function tests	Serum bilirubin	3.2 mg/dL (0.1-1.2 mg/dL)
	AST	225 U/L (8-33 U/L)
	ALT	270 U/L (4-36 U/L)
	Alkaline phosphatase	951 IU/L (44-147 IU/L)
Virology	HBV (ELISA)	Negative
	HCV (ELISA)	Negative
	HIV (ELISA)	Negative
Coagulation profile	D-dimers	1,150 ng/dL (<500 ng/dL)
	PT (INR)	31 seconds (1)
	aPTT	27 seconds (21-25 seconds)
	Platelets	191,000/µL
	Fibrinogen	1.8 g/dL (2-4 g/L)
	FDP	13 mcg/mL (<10 mcg/mL)

His electrocardiogram (ECG) showed normal sinus rhythm, and his chest X-ray showed bilateral pleural effusion with a normal cardiothoracic ratio, while his echocardiography revealed an intracardiac thrombus extending from the inferior vena cava (IVC) and a slightly dilated right atrium and superior and inferior vena cava. The ejection fraction of the right heart was slightly reduced to 48% with irregular flow through a tricuspid valve with incomplete closure.

Based on these findings, a diagnosis of HCC with a tumor thrombus extending to the right atrium was made. A multidisciplinary committee decided that the patient was not a candidate for surgical or ablative therapies. Thus, he was conservatively managed in our unit for heart failure and HCC but died on day 7 of admission, collapsing suddenly with dyspnea, tachypnea, tachycardia, and desaturation. The management of a suspected pulmonary embolism was unsuccessful and was considered the probable cause of his death; however, it was not confirmed.

## Discussion

Intracardiac metastasis of tumor associated with hepatocellular carcinoma (HCC) is a rare manifestation of hepatocellular carcinoma [7]. The two routes of spread of hepatocellular carcinoma into the heart are a continuous hematogenous spread of the tumor along the inferior vena cava into the heart and a direct spread of the HCC from various surrounding structures into the heart [8]. HCC associated with a tumor thrombus extending through the inferior vena cava up to the right atrium is uncommon with an incidence of 2.9% by imaging techniques, 0.7% at operation, and 18.2% at autopsy in Japan [9]. The mean survival of HCC-related thrombus formation and its extension to the right heart is three days to two months if left untreated or not diagnosed promptly. However, with surgery, the one-year survival is up to 40% in one study, which signifies the role of early intervention [10].

The signs and symptoms of HCC with tumor thrombus metastasis and heart failure often overlap; therefore, diagnosing intracardiac involvement can be difficult. However, this diagnostic delay can be prevented by performing early echocardiography to look for cardiac involvement in patients with a large tumor burden of HCC. A definitive diagnosis of a heart mass as metastatic HCC can be made by performing cardiac catheterization and obtaining a biopsy sample [11]. This may ultimately lead to the use of potentially useful procedures with survival benefits such as hypofractionated radiotherapy and surgery with embolectomy for HCC associated with tumor thrombus formation, which could improve the overall condition and improve progression-free survival of patients [10].

Different treatment modalities may be employed for intracardiac tumor thrombus extension depending on the extent of tumor spread, hepatic functional reserve, and underlying comorbidities of the patient. These include surgical resection with embolectomy, chemotherapy, transarterial chemoembolization (TACE), and radiofrequency ablation with a one-year survival benefit of 7%-18% in the study by Pesi et al. [12]. Surgical intervention has been proven useful in terms of survival benefit in preventing or treating acute life-threatening complications such as right ventricular outflow obstruction, valvular (tricuspid) compromise, and pulmonary embolism and used palliatively to reduce the severity of heart failure symptoms. Different surgical approaches can be used, although whichever to use is not standardized, but few approaches are widely practiced with good results. These include cardiopulmonary bypass (CPB) and total hepatic vascular exclusion (THVE). Utilizing the use of hypothermic cardiocirculatory arrest (HCCA) along with CPB has good results in terms of reducing blood loss and achieving hemostasis of the resected plane [12]. The survival of patients who underwent surgery for cardiac tumor thrombosis was 40% in one year compared to 0% without surgery [10].

The above-discussed treatment options have their pros and cons, and the decision on the appropriate treatment should be made individually based on the severity of the disease and underlying comorbidities of the patient. Further investigation into the optimal treatment of advanced HCC with cardiac metastases is warranted to increase overall and progression-free survival.

## Conclusions

HCC with tumor thrombus should be diagnosed early, and any patient with HCC who presents with emerging signs and symptoms of right heart failure should be evaluated urgently to diagnose metastasis, particularly tumor thrombus. Against this background, a cardiological and surgical consultation should be urgently advised. Tumor thrombus detected early, when confined to the hepatic vein, can be treated with better outcomes than when diagnosed at the time of cardiac metastasis. Therefore, further studies and work need to be done on whether screening diagnosed HCC patients for tumor thrombus metastases is necessary. Our team working on this article also suggested an additional intervention through the use of inferior vena cava filters (once the thrombus is confined to the hepatic vein), which could be used to prevent the further spread of the tumor thrombus and its adverse outcomes. However, it was difficult to use and evaluate in this resource-constrained setting.
